# Is Pleural Fluid Presepsin a Reliable Diagnostic Biomarker for Pleural Effusions?

**DOI:** 10.3390/diagnostics16193122

**Published:** 2026-09-25

**Authors:** Nihal Ertürk, Habibe Selmin Özensoy, Şervan Gökhan, Esra Fırat Oğuz, Funda Eren

**Affiliations:** 1 Emergency Department, Ankara Bilkent City Hospital, 06800 Ankara, Türkiye; habibeozensoy@gmail.com; 2 Department of Emergency Medicine, School of Medicine, Ankara Yıldırım Beyazıt University, 06800 Ankara, Türkiye; sgokhan@ybu.edu.tr; 3 Department of Medical Biochemistry, Ankara Bilkent City Hospital, 06800 Ankara, Türkiye; dr_esrafirat@hotmail.com; 4 Department of Medical Biochemistry, Istanbul Basaksehir Cam and Sakura City Hospital, 34000 Istanbul, Türkiye; fundakarakoyunlu@gmail.com

**Keywords:** pleural effusion, presepsin, Light’s criteria, biomarker, diagnostic accuracy, thoracentesis

## Abstract

**Background/Objectives:** Biochemical markers used in the diagnostic evaluation of pleural effusions are limited in both number and diagnostic accuracy. Presepsin has shown promise in bacterial infections. This study aimed to evaluate the diagnostic utility of pleural fluid presepsin for differentiating transudative from exudative pleural effusions. **Methods:** In this prospective observational study, presepsin concentrations were measured in pleural fluid obtained during diagnostic thoracentesis. Patients were classified according to Light’s criteria and final clinical diagnosis. Receiver operating characteristic (ROC) analysis was performed to assess diagnostic performance, while multivariable logistic regression identified independent predictors of exudative pleural effusions. Associations between presepsin concentrations and mortality, infectious etiology, and pleural fluid culture positivity were also analyzed. **Results:** Eighty-eight patients were included, comprising 48 (54.5%) exudative and 40 (45.5%) transudative pleural effusions. Median pleural fluid presepsin concentrations were significantly higher in transudative than in exudative effusions (410.79 vs. 368.64 pg/mL, *p* = 0.010). A conventional model based on routinely available clinical and laboratory variables yielded an AUC of 0.797, which increased to 0.807 after adding pleural fluid presepsin (ΔAUC = 0.010; DeLong *p* = 0.606). Pleural fluid presepsin showed limited discrimination for infectious effusions (AUC = 0.618) and provided no significant incremental diagnostic value beyond routine biomarkers. **Conclusions:** Pleural fluid presepsin showed limited diagnostic performance and did not provide incremental diagnostic information beyond routinely available biomarkers for either transudative–exudative or infectious–noninfectious classification. The unexpectedly higher concentrations in transudative effusions remain observational and cannot be attributed to a specific biological mechanism.

## 1. Introduction

Pleural effusion (PE) is a common clinical problem associated with various pulmonary and systemic disorders, including heart failure, pneumonia, malignancy, pulmonary embolism, liver cirrhosis, and inflammatory diseases [1,2,3,4]. Because treatment and prognosis vary according to the underlying etiology, early and accurate diagnosis is essential. Diagnostic thoracentesis and routine biochemical, microbiological, and cytological analyses remain central to the evaluation of unexplained pleural effusions [4,5]. Light’s criteria have been used as a reference method for distinguishing transudative and exudative pleural effusions for over fifty years; however, despite their sensitivity of approximately 98% in identifying exudates, their low specificity can lead to the misclassification of 20–30% of transudative effusions as exudates, particularly in heart failure patients receiving diuretic therapy [2,6,7,8,9].

Light’s criteria classify pleural effusions according to their pathophysiological mechanism but do not determine the underlying etiology, which may require additional laboratory tests, imaging, pleural biopsy, or thoracoscopy [1,2]. Consequently, several pleural fluid biomarkers have been investigated to improve etiological differentiation and clinical decision-making [4,10,11]. However, no single biomarker has demonstrated sufficient diagnostic performance to replace conventional pleural fluid analysis. Identifying biomarkers that provide additional diagnostic or prognostic information therefore remains an important area of pleural research [4,5,12].

Presepsin, also known as soluble CD14 subtype (sCD14-ST), is an approximately 13-kDa glycoprotein fragment generated during monocyte and macrophage activation following pathogen recognition and phagocytosis [13]. It has been investigated as a biomarker of bacterial infection and systemic innate immune activation. Systematic reviews and meta-analyses have demonstrated that presepsin has diagnostic and prognostic value in sepsis, with performance comparable or complementary to conventional inflammatory biomarkers such as procalcitonin and CRP [14,15]. Elevated presepsin concentrations have also been associated with disease severity and adverse outcomes in community-acquired pneumonia and other respiratory infections [16].

The role of presepsin in pleural diseases remains insufficiently understood. Previous studies have reported elevated pleural fluid presepsin concentrations in infectious effusions, particularly parapneumonic effusions and empyema, suggesting a potential role in distinguishing infectious from non-infectious pleural disease [17]. However, the available evidence is limited and has primarily focused on relatively small populations with infectious pleural effusions. Whether pleural fluid presepsin can differentiate transudative from exudative effusions, provide diagnostic information beyond conventional laboratory parameters and Light’s criteria, or predict clinically relevant outcomes such as 30-day mortality therefore remains uncertain [17,18,19]. Accordingly, this study aimed to evaluate the diagnostic performance of pleural fluid presepsin for transudative–exudative and infectious–noninfectious classification and to determine whether it provides incremental diagnostic value beyond routinely available biomarkers.

## 2. Materials and Methods

### 2.1. Study Design and Setting

This prospective observational diagnostic accuracy study was conducted in the emergency department of a tertiary university hospital between June 2024 and June 2026. The study was designed to evaluate the diagnostic utility of pleural fluid presepsin without affecting routine clinical decision-making processes. The study protocol was approved by the institutional ethics committee and conducted in accordance with the Declaration of Helsinki. Written informed consent was obtained from all participants prior to enrollment.

### 2.2. Study Population

#### 2.2.1. Inclusion and Exclusion Criteria

Adult patients (≥18 years) who underwent diagnostic thoracentesis for newly detected pleural effusion during the study period were screened consecutively for eligibility.

Patients were excluded if they

Were younger than 18 years;
Were pregnant;
Were unwilling or unable to provide written informed consent;
Had conditions preventing adequate pleural fluid sampling;
Died before simultaneous collection of pleural fluid and serum samples;
Died before a definitive etiological diagnosis could be established;
Had no final confirmed diagnosis explaining the pleural effusion.

Patients in whom a definitive diagnosis could not be established because they declined further diagnostic procedures (e.g., thoracoscopy or pleural biopsy) were documented separately and excluded from the primary diagnostic analyses.

#### 2.2.2. Patient Selection

**Figure 1:**
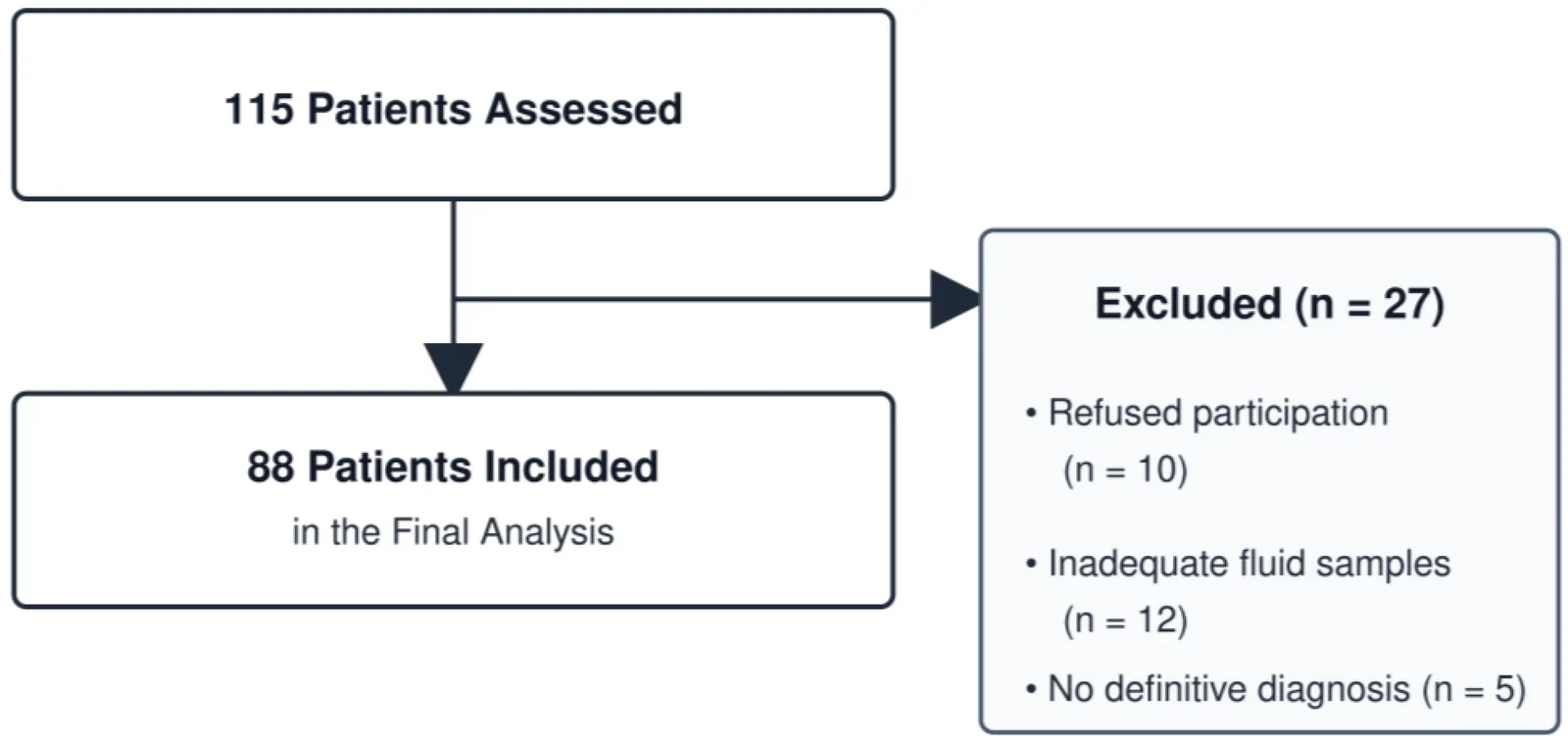
Study Flow Diagram.

A total of 115 consecutive patients who underwent diagnostic thoracentesis for pleural effusion were initially assessed for eligibility. Of these, 10 patients declined to participate, 12 were excluded because of inadequate pleural fluid samples for laboratory analysis, and 5 were excluded due to the absence of a definitive final diagnosis. Consequently, 88 patients fulfilled all eligibility criteria and were included in the final statistical analysis. The patient selection process and reasons for exclusion are summarized in Figure 1.

### 2.3. Clinical Management

All diagnostic and therapeutic decisions were made independently by the treating physicians according to current clinical practice. The results of pleural fluid presepsin measurements were not available to clinicians and therefore did not influence patient management.

Infectious pleural effusion was defined based on compatible clinical, radiological, biochemical, microbiological, and/or therapeutic findings and included parapneumonic effusions and empyema. The infectious effusion group included two patients with empyema. Because of the very small number of empyema cases, empyema was not analyzed as a separate diagnostic subgroup. Complicated parapneumonic effusion was not prospectively coded as a separate diagnostic category and could not be reliably reconstructed retrospectively.

### 2.4. Sample Collection and Laboratory Analysis

Pleural fluid samples were collected during clinically indicated diagnostic thoracentesis. An aliquot of pleural fluid was transferred into EDTA-containing tubes and stored at −80 °C until analysis. Blood samples for serum biochemical and inflammatory marker analyses were collected contemporaneously with thoracentesis whenever clinically feasible. Before presepsin measurement, samples were thawed and centrifuged at 1000× *g* for 15 min at 4 °C, and the supernatant was used for analysis.

Routine biochemical, microbiological, cytological, and hematological parameters were recorded simultaneously. Additional analyses were performed to assess the potential influence of renal function on pleural fluid presepsin concentrations. Light’s criteria were used as the reference method for classifying pleural effusions as transudative or exudative. An effusion was classified as exudative if at least one of the following criteria was met: (1) pleural fluid protein/serum protein ratio > 0.5; (2) pleural fluid LDH/serum LDH ratio > 0.6; or (3) pleural fluid LDH > two-thirds of the upper limit of normal serum LDH. Effusions meeting none of these criteria were classified as transudative.

Presepsin concentrations were measured using a commercially available human presepsin (PSPN) enzyme-linked immunosorbent assay (ELISA) kit (BT Lab, Bioassay Technology Laboratory, Jiaxing, China; catalog No. E3754Hu; lot No. 202512019), according to the manufacturer’s instructions. The assay had a sensitivity of 2.39 ng/L and a detection range of 5–1000 ng/L. The intra-assay and inter-assay coefficients of variation were <8% and <10%, respectively.

### 2.5. Study Outcomes

The primary outcome was the diagnostic performance of pleural fluid presepsin for differentiating exudative from transudative pleural effusions according to Light’s criteria. Secondary outcomes included the diagnostic performance of presepsin for infectious versus noninfectious pleural effusions, its comparison with established inflammatory and pleural biomarkers, its incremental diagnostic value when added to conventional models, and the relationship between pleural presepsin and renal function.

### 2.6. Statistical Analysis

Statistical analyses were performed using IBM SPSS Statistics for Windows, version 26.0 (IBM Corp., Armonk, NY, USA). Additional logistic regression models, ROC analyses, DeLong comparisons, and Holm-adjusted pairwise comparisons were performed using Python 3.12.14. Continuous variables were expressed as median (95% confidence interval [CI]) or median (interquartile range [IQR]), as specified in the corresponding tables. Two-group comparisons were performed using the Mann–Whitney U test, whereas comparisons involving three groups were performed using the Kruskal–Wallis test. When the overall Kruskal–Wallis test was statistically significant, pairwise comparisons were conducted using the Mann–Whitney U test with Holm adjustment for multiple testing. Categorical variables were presented as frequencies (percentages) and compared using the chi-square or Fisher’s exact test, as appropriate.

Spearman’s rank correlation analysis was used to evaluate correlations between biomarkers. ROC curve analysis was performed to determine the discriminative performance of biomarkers for identifying exudative pleural effusion. The area under the ROC curve (AUC) with 95% CIs, optimal cut-off values based on the Youden index, sensitivity, specificity, positive predictive value, and negative predictive value were calculated.

ROC analyses for infectious versus noninfectious pleural effusions were performed for pleural fluid presepsin, serum CRP, pleural fluid LDH, pleural fluid WBC, pleural fluid glucose, and serum procalcitonin. Infectious pleural effusion was designated as the positive outcome. AUCs were compared using DeLong’s test. Holm correction was applied to comparisons between presepsin and multiple conventional biomarkers.

For transudative–exudative classification, a conventional logistic regression model containing age, serum CRP, serum LDH, and eGFR was compared with the same model after addition of pleural fluid presepsin. For infectious effusion classification, serum CRP alone was compared with serum CRP plus presepsin. A multivariable routine biomarker model containing serum CRP, pleural fluid LDH, pleural fluid WBC, and pleural fluid glucose was also compared with the same model after addition of presepsin. Differences between model AUCs were evaluated using DeLong’s test.

Serum creatinine and eGFR were compared across Light’s classification and etiological groups. The proportion of patients with eGFR < 60 mL/min/1.73 m^2^ was also calculated. Associations between pleural presepsin and creatinine or eGFR were evaluated using Spearman’s correlation. To examine whether the association between effusion type and presepsin was independent of renal function and other potential confounders, log-transformed pleural presepsin was modeled as the dependent variable, with effusion type, eGFR, age, serum CRP, and volume overload as covariates.

## 3. Results

### 3.1. Characteristics

A total of 88 patients with pleural effusion were included. The median age was 71.5 years (95% CI, 66.0–76.0), and 41 patients (46.6%) were male. According to Light’s criteria, 40 patients (45.5%) had transudative and 48 (54.5%) had exudative pleural effusions. Pleural fluid culture was available for 71 patients, of whom 10 (14.1%) had positive culture results. The median pleural fluid presepsin concentration was 391.54 pg/mL (95% CI, 375.45–410.79). The baseline demographic, clinical, and laboratory characteristics are summarized in Table 1.

### 3.2. Laboratory Findings by Etiology

Laboratory findings according to the etiological diagnosis are presented in Table 2. Serum CRP, pleural fluid protein, pleural fluid LDH, pleural fluid WBC, and pleural fluid glucose differed significantly among the volume overload, infectious, and malignant pleural effusion groups. In Holm-adjusted post-hoc comparisons, the volume overload group had significantly lower serum CRP, pleural fluid protein, and pleural fluid LDH concentrations and higher pleural fluid glucose concentrations than the infectious and malignant groups. Although the overall difference in pleural fluid WBC was statistically significant, none of the pairwise comparisons remained significant after adjustment for multiple testing. Pleural fluid presepsin concentrations did not differ significantly among the three etiological groups (*p* = 0.064). Renal function differed significantly across etiological groups. The volume overload group had higher creatinine and lower eGFR values than the infectious and malignant groups. An eGFR below 60 mL/min/1.73 m^2^ was present in 60.5% of the volume overload group, 35.7% of the infectious group, and 13.6% of the malignant group (Table 2).

### 3.3. Pleural Fluid Presepsin According to Clinical Characteristics

Pleural fluid presepsin concentrations were significantly higher in transudative than in exudative pleural effusions (410.79 vs. 368.64 pg/mL, *p* = 0.010). Pleural fluid presepsin concentrations differed significantly across hospital disposition categories (*p* = 0.004). However, pleural fluid presepsin concentrations did not differ significantly according to pleural fluid culture results, final diagnosis, or 30-day mortality. Transudative effusions had higher serum creatinine values than exudative effusions [1.12 (0.80–1.65) vs. 0.80 (0.68–1.19) mg/dL; *p* = 0.008] and lower eGFR values [53.0 (35.8–72.8) vs. 84.5 (55.0–102.3) mL/min/1.73 m^2^; *p* = 0.002]. An eGFR below 60 mL/min/1.73 m^2^ was observed in 55.0% of transudative and 29.2% of exudative effusions (*p* = 0.014; Table 3).

### 3.4. Incremental Diagnostic Value of Pleural Fluid Presepsin for Transudative–Exudative Classification

The conventional model including age, serum CRP, serum LDH, and eGFR yielded an AUC of 0.797 (95% CI 0.701–0.894). Addition of pleural fluid presepsin increased the AUC to 0.807 (95% CI 0.716–0.899); however, this improvement was not statistically significant (ΔAUC = 0.010; DeLong *p* = 0.606) (Table 4).

### 3.5. Diagnostic Performance for Differentiating Exudative and Transudative Pleural Effusions

**Figure 2:**
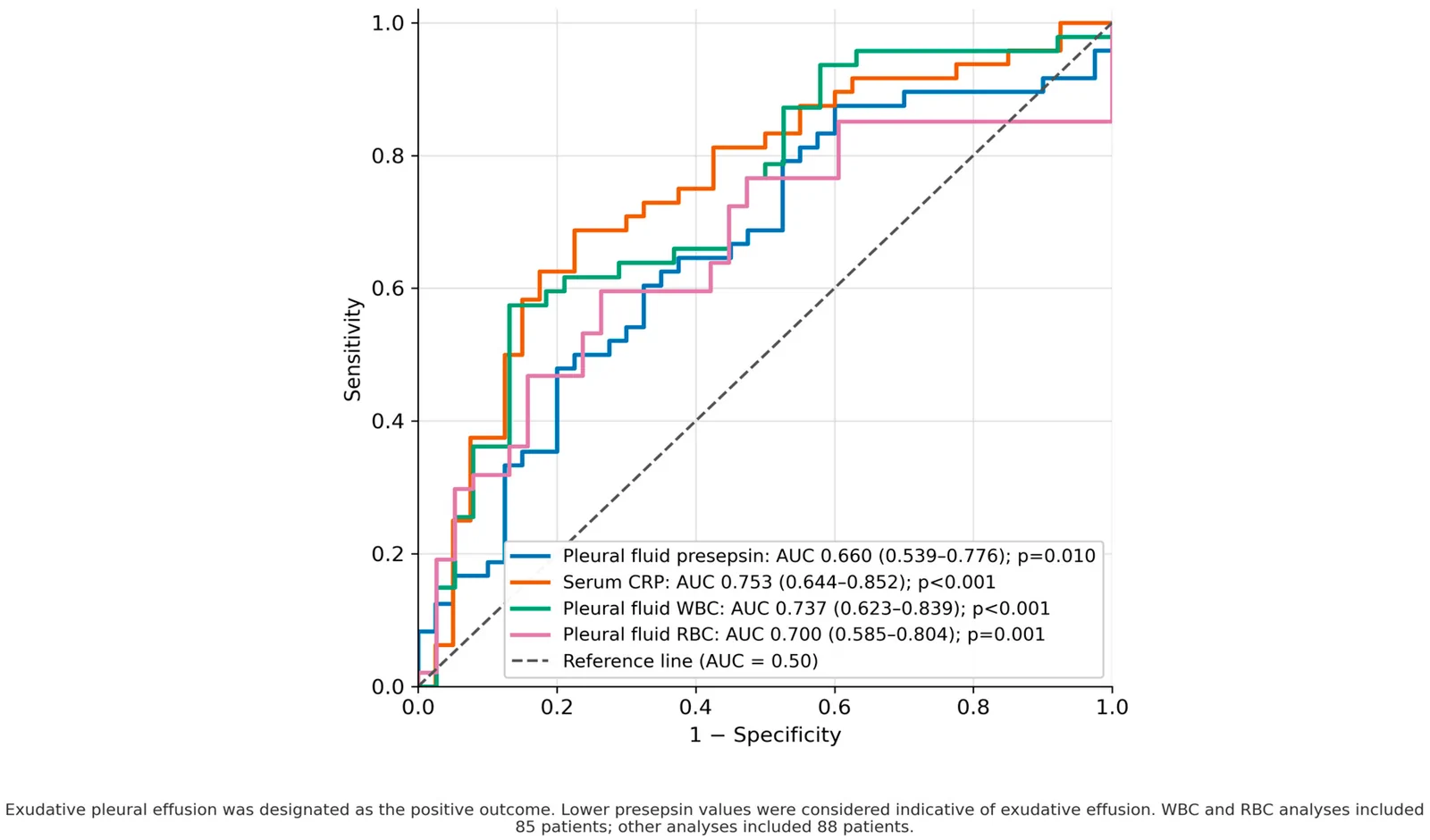
ROC Curves for Selected Biomarkers in Differentiating Exudative From Transudative Pleural Effusions.

ROC analysis was performed to evaluate the ability of pleural fluid presepsin and conventional biomarkers to differentiate exudative from transudative pleural effusions (Table 5 and Figure 2). Serum CRP demonstrated the highest discriminatory performance (AUC, 0.753; 95% CI, 0.644–0.852; *p* < 0.001), followed by pleural fluid WBC (AUC, 0.737; 95% CI, 0.623–0.839; *p* < 0.001) and pleural fluid RBC (AUC, 0.700; 95% CI, 0.585–0.804; *p* = 0.001). When lower presepsin concentrations were considered indicative of exudative effusion, pleural fluid presepsin demonstrated modest discriminatory performance, with an AUC of 0.660 (95% CI, 0.539–0.776; *p* = 0.010). A presepsin cut-off of ≤387.22 pg/mL provided 60.4% sensitivity, 67.5% specificity, a positive predictive value of 69.0%, and a negative predictive value of 58.7%. Serum procalcitonin, pleural fluid glucose, and pleural fluid sodium did not demonstrate statistically significant discriminatory performance.

### 3.6. Added Value for Infectious EffusionClassification

Serum CRP alone yielded an AUC of 0.759, compared with 0.760 after addition of pleural fluid presepsin (ΔAUC = 0.001; DeLong *p* = 0.937). Similarly, the routine biomarker model yielded an AUC of 0.761, compared with 0.759 after addition of presepsin (ΔAUC = −0.002; DeLong *p* = 0.715). Presepsin was not independently associated with infectious effusion in either model (Table 6).

### 3.7. Diagnostic Performance for Infectious Effusions

Twenty-eight patients were classified as having infectious pleural effusions and 60 as having noninfectious effusions. Two patients with empyema were included in the infectious group. Pleural fluid presepsin showed limited discrimination for infectious effusions, with an AUC of 0.618 (95% CI 0.482–0.754). A cut-off of ≤360.77 pg/mL yielded 60.7% sensitivity and 73.3% specificity. Serum CRP and pleural fluid LDH demonstrated higher numerical AUCs of 0.759 and 0.726, respectively (Table 7). However, none of the pairwise differences remained statistically significant after Holm correction (adjusted *p* = 0.435 and *p* = 0.583, respectively).

### 3.8. Correlation Analysis

Spearman correlation analysis demonstrated weak but statistically significant negative correlations between pleural fluid presepsin concentrations and serum CRP (ρ = −0.227, *p* = 0.034), pleural fluid LDH (ρ = −0.290, *p* = 0.006), and Light’s criteria (ρ = −0.276, *p* = 0.009). No significant correlation was observed between pleural fluid presepsin concentration and 30-day mortality (ρ = −0.113, *p* = 0.296). No significant correlations were observed between pleural presepsin and creatinine (ρ = 0.107; *p* = 0.321) or eGFR (ρ = −0.107; *p* = 0.320) (Table 8).

### 3.9. Renal Function-Adjusted Analysis of Pleural Fluid Presepsin

In the model adjusted for eGFR, age, and serum CRP, the association between effusion type and log-transformed pleural fluid presepsin was borderline (β = −0.150, 95% CI −0.305 to 0.004; *p* = 0.056). After additional adjustment for volume overload, lower log-transformed presepsin remained associated with exudative status (β = −0.188, 95% CI −0.369 to −0.008; *p* = 0.041). The variation between these models warrants cautious interpretation.

## 4. Discussion

### 4.1. Summary of Findings

Pleural fluid presepsin showed modest discrimination for transudative–exudative classification and limited discrimination for infectious effusions. Its addition to conventional or multivariable routine biomarker models did not significantly improve diagnostic performance. Serum CRP and pleural fluid LDH showed better numerical performance for identifying infectious effusions. Renal function differed significantly across Light and etiological groups, although pleural presepsin was not significantly correlated with creatinine or eGFR.

### 4.2. Comparison with Previous Studies

Interpretation of our findings should consider the extremely limited evidence regarding pleural fluid presepsin. Only a limited number of studies have specifically evaluated presepsin concentrations in pleural fluid. A previous study by Watanabe et al. reported significantly higher pleural fluid presepsin concentrations in empyema and parapneumonic effusions than in non-infectious pleural effusions [17]. In our cohort, only two patients had empyema, and both were included in the infectious effusion group. The limited number of empyema cases precluded a reliable separate ROC analysis. Moreover, complicated parapneumonic effusion was not prospectively coded as a separate diagnostic category. Consequently, direct comparison between the two studies is limited by substantial differences in study populations and disease distribution.

Previous studies have reported that circulating presepsin concentrations may be influenced by renal dysfunction, heart failure, and systemic inflammatory conditions [20,21,22,23,24,25]. In the present study, the unexpectedly higher pleural fluid presepsin concentrations observed in transudative effusions should be considered hypothesis-generating. Renal function differed significantly between effusion and etiological groups, particularly in the volume overload group. However, pleural fluid presepsin was not significantly correlated with either serum creatinine or eGFR, and the association between effusion type and presepsin varied across adjusted models. Therefore, renal dysfunction alone does not fully explain the observed pattern. Differences in age, systemic inflammation, volume overload, and other etiological characteristics may also have contributed. These findings do not establish a specific biological mechanism. Because paired serum presepsin measurements were not obtained, it remains unclear whether pleural fluid presepsin reflects local pleural production, passive transfer from the circulation, or systemic presepsin concentrations.

### 4.3. Prognostic Value

Unlike previous studies demonstrating that elevated circulating presepsin concentrations are associated with adverse outcomes and increased mortality in patients with sepsis, acute heart failure, and other critically ill populations, pleural fluid presepsin concentrations were not associated with 30-day mortality in our cohort [25,26,27,28].

### 4.4. Clinical Implications

Our findings suggest that pleural fluid presepsin should not be used as a stand-alone biomarker for differentiating transudative from exudative pleural effusions. Instead, its interpretation should always be integrated with clinical findings, Light’s criteria, radiological evaluation, and conventional pleural fluid analyses. In particular, elevated pleural fluid presepsin concentrations in patients with heart failure should not automatically be interpreted as evidence of pleural infection, since systemic inflammation, renal dysfunction, and etiological differences may contribute to the observed presepsin distribution. Pleural fluid presepsin should also not be used as a stand-alone marker for infectious pleural effusion because its discrimination was limited and it did not improve models containing conventional biomarkers.

### 4.5. Strengths

The present study has several strengths. To our knowledge, it is one of the first studies investigating pleural fluid presepsin across a broad spectrum of pleural effusion etiologies. In addition, all patients underwent comprehensive routine clinical evaluation, reducing the risk of diagnostic misclassification. Finally, the simultaneous assessment of diagnostic accuracy, prognostic value, and correlations with conventional biomarkers provides a multifaceted evaluation of the potential clinical utility of pleural fluid presepsin.

### 4.6. Limitations

Several limitations should be acknowledged. This was a single-center study with a relatively small sample size, limiting the generalizability of the findings. Detailed comorbidity data, including prospectively adjudicated AKI and CKD status, were not collected. However, serum creatinine and eGFR were available for all participants and were incorporated into the renal function analyses. Residual confounding related to renal and cardiac dysfunction may nevertheless remain. Serum presepsin concentrations were not measured simultaneously, preventing direct comparison between systemic and pleural fluid presepsin levels. Detailed procedural data regarding pleuroscopy, PET imaging, pleural biopsy, and VATS were not prospectively collected in a standardized manner; therefore, procedure-specific diagnostic analyses could not be performed. Pleural neutrophil count or percentage was unavailable, preventing direct comparison with this established marker. Only two patients had empyema, and complicated parapneumonic effusion was not prospectively coded separately, precluding reliable subgroup-specific diagnostic analyses. Finally, serial measurements were not available, precluding assessment of dynamic changes during treatment, and residual confounding cannot be completely excluded because of the observational study design.

## 5. Conclusions

Pleural fluid presepsin showed limited diagnostic performance for both transudative–exudative and infectious–noninfectious classification. It did not significantly improve discrimination when added to conventional or multivariable routine biomarker models. Serum CRP and pleural fluid LDH showed better numerical performance for identifying infectious effusions. The unexpectedly higher presepsin concentrations in transudative effusions remain an observational finding and cannot be attributed to local pleural production or a specific biological mechanism without paired serum presepsin measurements. In conclusion, pleural fluid presepsin should not currently be used as a stand-alone diagnostic biomarker in patients with pleural effusion.

## Figures and Tables

**Table 1:** Baseline Demographic, Clinical, and Laboratory Characteristics of the Study Population (*n* = 88).

Variable	Overall (*n* = 88)
Demographic characteristics	
Age, years	71.5 (66.0–76.0)
Female sex, *n* (%)	47 (53.4)
Male sex, *n* (%)	41 (46.6)
Pleural effusion characteristics	
Transudative pleural effusion, *n* (%)	40 (45.5)
Exudative pleural effusion, *n* (%)	48 (54.5)
Positive pleural fluid culture, *n*/N (%)	10/71 (14.1)
Final diagnosis	
Volume overload, *n* (%)	38 (43.2)
Infectious pleural effusion, *n* (%)	28 (31.8)
Malignant pleural effusion, *n* (%)	22 (25.0)
Clinical outcomes	
Discharged from hospital, *n* (%)	36 (40.9)
Ward admission, *n* (%)	24 (27.3)
Intensive care unit admission, *n* (%)	28 (31.8)
30-day mortality, *n* (%)	26 (29.5)
Laboratory findings	
Pleural fluid presepsin, pg/mL	391.54 (375.45–410.79)
Serum protein, g/L	62.0 (60.0–64.0)
Serum LDH, U/L	270.5 (236.0–304.0)
Serum CRP, mg/L	36.0 (26.0–56.0)
Serum procalcitonin, ng/mL	0.10 (0.10–0.15)
Pleural fluid protein, g/L	31.0 (29.0–34.0)
Pleural fluid LDH, U/L	117.0 (107.0–182.0)
Pleural fluid leukocyte count, /µL	203 (146–358)
Pleural fluid erythrocyte count, ×10^3^/µL	5 (4–10)
Pleural fluid glucose, mg/dL	116.5 (108.0–130.0)
Pleural fluid sodium, mmol/L	141.0 (140.0–143.0)
Serum creatinine, mg/dL	0.98 (0.70–1.40)
eGFR, mL/min/1.73 m^2^	69.5 (44.8–93.3)
eGFR < 60 mL/min/1.73 m^2^, *n* (%)	36 (40.9)

Continuous variables are presented as median (95% confidence interval for the median), except for serum creatinine and eGFR, which are presented as median (interquartile range). Categorical variables are presented as *n* (%). Pleural fluid leukocyte and erythrocyte counts were available for 85 patients, and pleural fluid culture results were available for 71 patients. CRP, C-reactive protein; eGFR, estimated glomerular filtration rate; LDH, lactate dehydrogenase.

**Table 2:** Laboratory Findings According to Etiological Diagnosis.

Laboratory Parameter	Volume Overload (*n* = 38)	Infectious (*n* = 28)	Malignant (*n* = 22)	*p* Value
Pleural fluid presepsin, pg/mL	410.79 (369.52–452.03)	354.34 (322.99–420.01)	381.84 (333.07–416.96)	0.064
Serum protein, g/L	61.50 (56.00–67.75)	62.00 (57.00–68.25)	61.50 (55.25–65.75)	0.752
Serum LDH, U/L	259.00 (195.50–309.00)	295.00 (214.75–398.50)	278.00 (225.50–342.25)	0.107
Serum CRP, mg/L	14.00 (7.85–36.00)	131.00 (39.50–182.25)	51.50 (25.80–113.50)	**<0.001**
Serum procalcitonin, ng/mL	0.09 (0.06–0.15)	0.10 (0.06–0.26)	0.10 (0.06–0.16)	0.676
Pleural fluid protein, g/L	23.50 (18.45–31.00)	32.00 (24.00–46.25)	35.00 (29.75–45.50)	**<0.001**
Pleural fluid LDH, U/L	95.00 (76.25–112.00)	245.50 (131.75–678.25)	285.50 (134.00–505.50)	**<0.001**
Pleural fluid WBC, /µL	141.50 (85.25–327.50)	512.00 (144.00–1200.00)	360.00 (121.50–1525.00)	**0.047**
Pleural fluid RBC, ×10^3^/µL	5.00 (0.00–10.00)	4.00 (0.50–68.00)	10.00 (3.25–36.00)	0.102
Pleural fluid glucose, mg/dL	131.50 (109.50–176.25)	108.00 (98.00–131.25)	99.00 (72.25–134.25)	**0.010**
Pleural fluid sodium, mmol/L	142.00 (140.00–144.75)	139.50 (136.00–144.00)	140.00 (136.50–143.75)	0.193
Serum creatinine mg/dL	1.18 (0.83–1.88)	0.86 (0.60–1.29)	0.75 (0.70–0.97)	**0.003**
eGFR	50.5 (29.0–71.8)	73.5 (54.0–108.0)	89.0 (73.3–100.8)	**<0.001**
EGFR <60 *n* (%)	23 (60.5%)	10 (35.7%)	3 (13.6%)	**0.001**

Data are presented as median (interquartile range). Overall comparisons were performed using the Kruskal–Wallis test. Pleural fluid WBC and RBC measurements were available for 85 patients. Statistically significant *p* values are shown in bold. CRP, C-reactive protein; LDH, lactate dehydrogenase; RBC, red blood cell count; WBC, white blood cell count.

**Table 3:** Pleural Fluid Presepsin Levels According to Clinical Characteristics.

Variable	Category	Pleural Fluid Presepsin, Median (95% CI), pg/mL	*p* Value
Light’s classification	Transudate	410.79 (387.23–433.80)	**0.010**
	Exudate	368.64 (336.68–404.47)	
Pleural fluid culture	Negative	389.37 (366.29–420.22)	0.710
	Positive	406.36 (317.40–430.29)	
Final diagnosis	Volume overload	410.79 (373.13–431.52)	0.064
	Infectious pleural effusion	354.34 (328.41–421.05)	
	Malignant pleural effusion	381.84 (340.72–425.38)	
Hospital disposition	Discharged	410.24 (373.46–447.87)	**0.004**
	Ward admission	354.34 (294.06–367.74)	
	Intensive care unit (ICU) admission	410.24 (379.03–441.15)	
30-day mortality	Survivors	408.57 (364.58–417.54)	0.293
	Non-survivors	367.61 (345.49–420.85)	
Creatinine mg/dL	Transudate	1.12 (0.80–1.65)	**0.008**
	Exudate	0.80 (0.68–1.19)	
eGFR mL/dk/1.73 m^2^	Transudate	53.0 (35.8–72.8)	**0.002**
	Exudate	84.5 (55.0–102.3)	
eGFR < 60	Transudate	22 (55.0%)	**0.014**
	Exudate	14 (29.2%)	

Pleural fluid presepsin concentrations are presented as median (95% confidence interval for the median); serum creatinine and eGFR are presented as median (interquartile range); and eGFR < 60 mL/min/1.73 m^2^ is presented as *n* (%). *p* values were calculated using the Mann–Whitney U test or Kruskal–Wallis test, as appropriate, except for eGFR < 60 mL/min/1.73 m^2^, which was compared using the chi-square test. Pleural fluid culture results were available for 71 patients. Statistically significant *p* values are shown in bold. CI, confidence interval; eGFR, estimated glomerular filtration rate.

**Table 4:** Incremental Value of Pleural Fluid Presepsin for Transudative–Exudative Classification.

Model	AUC (95% CI)	ΔAUC	DeLong *p*
Conventional model	0.797 (0.701–0.894)	Reference	—
Conventional model + pleural fluid presepsin	0.807 (0.716–0.899)	+0.010	0.606

The conventional model included age, serum CRP, serum LDH, and eGFR. AUC, area under the receiver operating characteristic curve; CI, confidence interval; eGFR, estimated glomerular filtration rate.

**Table 5:** Diagnostic Performance of Pleural Fluid Presepsin and Conventional Biomarkers for Differentiating Exudative From Transudative Pleural Effusions.

Biomarker	Optimal Cut-Off	AUC (95% CI)	Sensitivity (%)	Specificity (%)	Youden Index	PPV (%)	NPV (%)	*p* Value
Pleural fluid presepsin, pg/mL	≤387.22	0.660 (0.539–0.776)	60.4	67.5	0.279	69.0	58.7	0.010
Serum procalcitonin, ng/mL	≤0.045	0.526 (0.406–0.650)	18.8	92.5	0.113	75.0	48.7	0.675
Serum CRP, mg/L	≥39.00	0.753 (0.644–0.852)	68.8	77.5	0.463	78.6	67.4	<0.001
Pleural fluid WBC, /µL	≥422.50	0.737 (0.623–0.839)	57.4	86.8	0.443	84.4	62.3	<0.001
Pleural fluid RBC, ×10^3^/µL	≥7.00	0.700 (0.585–0.804)	59.6	73.7	0.333	73.7	59.6	0.001
Pleural fluid glucose, mg/dL	≤103.00	0.594 (0.477–0.710)	43.8	87.5	0.313	80.8	56.5	0.132
Pleural fluid sodium, mmol/L	≤142.50	0.605 (0.490–0.721)	70.8	47.5	0.183	61.8	57.6	0.092

Exudative pleural effusion was designated as the positive outcome. Cut-off directions were selected according to the observed direction of association, and optimal cut-offs were identified using the maximum Youden index. Lower values of pleural fluid presepsin, serum procalcitonin, pleural fluid glucose, and pleural fluid sodium were considered indicative of exudative effusion, whereas higher serum CRP, pleural fluid WBC, and pleural fluid RBC values were considered indicative of exudative effusion. Pleural fluid WBC and RBC measurements were available for 85 patients; the remaining analyses included 88 patients. AUC, area under the receiver operating characteristic curve; CI, confidence interval; CRP, C-reactive protein; NPV, negative predictive value; PPV, positive predictive value; RBC, red blood cell count; WBC, white blood cell count.

**Table 6:** Incremental value of presepsin for infectious effusion classification.

Model	AUC	ΔAUC	DeLong *p*	Presepsin *p*
Serum CRP	0.759	Reference	—	—
CRP + presepsin	0.760	+0.001	0.937	0.752
Routine biomarker model	0.761	Reference	—	—
Routine model + presepsin	0.759	−0.002	0.715	0.920

**Table 7:** Diagnostic Performance for Infectious Pleural Effusions.

NPV (%)	PPV (%)	Specificity (%)	Sensitivity (%)	Cut-Off	AUC (95% CI)	Youden Index	Marker
80.0	51.5	73.3	60.7	≤360.77	0.618 (0.482–0.754)	0.340	Pleural fluid presepsin, pg/mL *
84.2	61.3	80.0	67.9	≥76	0.759 (0.642–0.877)	0.479	Serum CRP, mg/L
87.0	52.4	66.7	78.6	≥122	0.726 (0.609–0.844)	0.452	Pleural fluid LDH, U/L
78.2	50.0	74.1	55.6	≥460	0.617 (0.482–0.752)	0.297	Pleural fluid WBC, /µL
77.6	43.6	63.3	60.7	≤109	0.605 (0.481–0.729)	0.240	Pleural fluid glucose, mg/dL *
72.7	45.5	80.0	35.7	≥0.20	0.558 (0.425–0.691)	0.157	Serum procalcitonin, ng/mL

Infectious pleural effusion was designated as the positive outcome. * Lower pleural fluid presepsin and glucose values were considered indicative of infectious effusion. Pleural fluid WBC was available for 85 patients; the other analyses included 88 patients. AUC, area under the receiver operating characteristic curve; CI, confidence interval; CRP, C-reactive protein; LDH, lactate dehydrogenase; PPV, positive predictive value; NPV, negative predictive value; WBC, white blood cell count.

**Table 8:** Correlation Between Pleural Fluid Presepsin and Clinical Variables.

Variable	Spearman’s ρ	*p* Value
Serum CRP	−0.227	**0.034**
Pleural fluid LDH	−0.290	**0.006**
Light’s criteria	−0.276	**0.009**
30-day mortality	−0.113	0.296
Creatinine	0.107	0.321
eGFR	−0.107	0.320

Data were analyzed using Spearman’s rank correlation coefficient (ρ). Statistically significant *p* values are shown in bold. Light’s criteria classification was coded as 0 for transudative and 1 for exudative effusion.

## Data Availability

The datasets generated and analyzed during the current study are available from the corresponding author upon reasonable request.

## Abbreviations

The following abbreviations are used in this manuscript:

Abbreviation	Definition
ADA	Adenosine deaminase
AUC	Area under the curve
CI	Confidence interval
CRP	C-reactive protein
EDTA	Ethylenediaminetetraacetic acid
ICU	Intensive care unit
LDH	Lactate dehydrogenase
NPV	Negative predictive value
OR	Odds ratio
PE	Pleural effusion
PPV	Positive predictive value
RBC	Red blood cell
ROC	Receiver operating characteristic
sCD14-ST	Soluble CD14 subtype
SPSS	Statistical Package for the Social Sciences
suPAR	Soluble urokinase plasminogen activator receptor
U/L	Units per liter
WBC	White blood cell

## References

[1] Marel M., Zrůstová M., Stasný B., Light R.W. (1993). The incidence of pleural effusion in a well-defined region: Epidemiologic study in central Bohemia. Chest.

[2] Bhatnagar R., Maskell N. (2015). The modern diagnosis and management of pleural effusions. BMJ.

[3] Saguil A., Wyrick K., Hallgren J. (2014). Diagnostic approach to pleural effusion. Am. Fam. Physician.

[4] Arrigo C., Aloisio E., Rovegno L., Dolci A., Panteghini M. (2023). The laboratory investigation of pleural fluids: An update based on the available evidence. Ann. Clin. Biochem..

[5] Roberts M.E., Rahman N.M., Maskell N.A., Bibby A.C., Blyth K.G., Corcoran J.P., Edey A., Evison M., de Fonseka D., Hallifax R. (2023). British Thoracic Society Guideline for pleural disease. Thorax.

[6] Light R.W., Macgregor M.I., Luchsinger P.C., Ball W.C. (1972). Pleural effusions: The diagnostic separation of transudates and exudates. Ann. Intern. Med..

[7] Mohan G., Bhide P., Agrawal A., Kaul V., Chaddha U. (2023). A practical approach to pseudoexudative pleural effusions. Respir. Med..

[8] Bielsa S., Porcel J.M., Castellote J., Mas E., Esquerda A., Light R.W. (2012). Solving the Light’s criteria misclassification rate of cardiac and hepatic transudates. Respirology.

[9] Porcel J.M. (2010). Pleural effusions from congestive heart failure. Seminars in Respiratory and Critical Care Medicine.

[10] Chan C., Chan K.K.P. (2024). Pleural fluid biomarkers: A narrative review. J. Thorac. Dis..

[11] Porcel J.M. (2018). Biomarkers in the diagnosis of pleural diseases: A 2018 update. Ther. Adv. Respir. Dis..

[12] Sundaralingam A., Grabczak E.M., Burra P., Costa M.I., George V., Harriss E., Jankowska E.A., Janssen J.P., Karpathiou G., Laursen C.B. (2024). ERS statement on benign pleural effusions in adults. Eur. Respir. J..

[13] Yaegashi Y., Shirakawa K., Sato N., Suzuki Y., Kojika M., Imai S., Takahashi G., Miyata M., Furusako S., Endo S. (2005). Evaluation of a newly identified soluble CD14 subtype as a marker for sepsis. J. Infect. Chemother..

[14] Kondo Y., Umemura Y., Hayashida K., Hara Y., Aihara M., Yamakawa K. (2019). Diagnostic value of procalcitonin and presepsin for sepsis in critically ill adult patients: A systematic review and meta-analysis. J. Intensive Care.

[15] Wu C.C., Lan H.M., Han S.T., Chaou C.H., Yeh C.F., Liu S.H., Li C.-H., Blaney G.N., Liu Z.-Y., Chen K.-F. (2017). Comparison of diagnostic accuracy in sepsis between presepsin, procalcitonin, and C-reactive protein: A systematic review and meta-analysis. Ann. Intensive Care.

[16] Liu B., Yin Q., Chen Y.X., Zhao Y.Z., Li C.S. (2014). Role of presepsin (sCD14-ST) and the CURB65 scoring system in predicting severity and outcome of community-acquired pneumonia in an emergency department. Respir. Med..

[17] Watanabe N., Ishii T., Kita N., Kanaji N., Nakamura H., Nanki N., Ueda Y., Tojo Y., Kadowaki N., Bandoh S. (2018). The usefulness of pleural fluid presepsin, C-reactive protein, and procalcitonin in distinguishing different causes of pleural effusions. BMC Pulm. Med..

[18] Hooper C., Lee Y.C.G., Maskell N. (2010). BTS Pleural Disease Guideline Group. Investigation of a unilateral pleural effusion in adults: British Thoracic Society Pleural Disease Guideline 2010. Thorax.

[19] Light R.W. (2013). The Light criteria: The beginning and why they are useful 40 years later. Clin. Chest Med..

[20] Bıyık İ., Turhan Çağlar F.N., Işıksaçan N., Şahin M.H., Karabulut D., Katkat F., Aktürk F. (2018). Serum presepsin levels in patients with decompensated heart failure. J. Updates Cardiovasc. Med..

[21] Kouroupis D., Zografou I., Doukelis P., Patoulias D., Popovic D.S., Karakasis P., Pyrpasopoulou A., Stavropoulos K., Papadopoulos C., Giouleme O. (2025). Presepsin: An emerging biomarker in the management of cardiometabolic disorders. J. Pers. Med..

[22] Ishii J., Takahashi H., Nishimura H., Fujiwara W., Ohta M., Kawai H., Muramatsu T., Harada M., Yamada A., Naruse H. (2020). Circulating presepsin (soluble CD14 subtype) as a novel marker of mortality in patients treated at medical cardiac intensive care units. Eur. Heart J..

[23] Murphy S.P., Kakkar R., McCarthy C.P., Januzzi J.L. (2020). Inflammation in heart failure: JACC State-of-the-Art Review. J. Am. Coll. Cardiol..

[24] Nagata T., Yasuda Y., Ando M., Abe T., Katsuno T., Kato S., Tsuboi N., Matsuo S., Maruyama S. (2015). Clinical impact of kidney function on presepsin levels. PLoS ONE.

[25] Miyoshi M., Inoue Y., Nishioka M., Ikegame A., Nakao T., Kishi S., Doi T., Nagai K. (2019). Clinical evaluation of presepsin considering renal function. PLoS ONE.

[26] Yang H.S., Hur M., Yi A., Kim H., Lee S., Kim S.N. (2018). Prognostic value of presepsin in adult patients with sepsis: Systematic review and meta-analysis. PLoS ONE.

[27] Molano-Franco D., Arevalo-Rodriguez I., Muriel A., del Campo-Albendea L., Fernández-García S., Alvarez-Méndez A., Simancas-Racines D., Viteri A., Sanchez G., Fernandez-Felix B. (2023). Basal procalcitonin, C-reactive protein, interleukin-6, and presepsin for prediction of mortality in critically ill septic patients: A systematic review and meta-analysis. Diagn. Progn. Res..

[28] Velissaris D., Zareifopoulos N., Karamouzos V., Karanikolas E., Pierrakos C., Koniari I., Karanikolas M. (2021). Presepsin as a diagnostic and prognostic biomarker in sepsis. Cureus.

